# Transcriptional and Epigenetic Regulation of Cardiac Electrophysiology

**DOI:** 10.1007/s00246-019-02160-w

**Published:** 2019-07-25

**Authors:** Jesus Jimenez, Stacey L. Rentschler

**Affiliations:** Cardiovascular Division, Department of Medicine, and Department of Developmental Biology, Washington University School of Medicine, 309 McDonnell Science Bldg, 660 South Euclid Ave, Campus Box 8103, St Louis, MO 63110 USA

**Keywords:** Electrophysiology, *HEY2*, *KCNIP2*, Epigenetics, Histone modification

## Abstract

Spatiotemporal gene expression during cardiac development is a highly regulated process. Activation of key signaling pathways involved in electrophysiological programming, such as Notch and Wnt signaling, occurs in early cardiovascular development and these pathways are reactivated during pathologic remodeling. Direct targets of these signaling pathways have also been associated with inherited arrhythmias such as Brugada syndrome and arrhythmogenic cardiomyopathy. In addition, evidence is emerging from animal models that reactivation of Notch and Wnt signaling during cardiac pathology may predispose to acquired arrhythmias, underscoring the importance of elucidating the transcriptional and epigenetic effects on cardiac gene regulation. Here, we highlight specific examples where gene expression dictates electrophysiological properties in both normal and diseased hearts.

## Introduction

The human heart starts beating spontaneously about 3 weeks after fertilization [1]. To reach this remarkable milestone, embryonic cells must differentiate into various cardiac cell types and maintain their identity through regulated spatiotemporal gene expression [2–5]. Evidence is emerging that differential susceptibility to cardiac arrhythmias may arise from these early embryonic programming events, mediated in part through chamber and regional-specific enhancer activity and chromatin patterning [6]. Key mechanisms of transcriptional and epigenetic regulation of cardiovascular development and pathological states include post-translational histone modification, non-coding RNA, and DNA methylation. Here, we will highlight how chamber-specific transcriptional responses and histone modifications collectively function to activate or repress transcriptional activity and ultimately direct gene expression, which contributes to chamber specificity in electrical homeostasis, as well as electrical “remodeling” in disease.

## Theory of Metabolic Memory

Modifications to the nucleosome, the basic unit of chromatin consisting of DNA coiled around histones, controls packaging of DNA and regulates access of transcription factors to the DNA [7]. There are at least 15 types of functional histone modifications which can occur, and the most commonly studied modifications in the heart are histone methylation/demethylation and acetylation/deacetylation [4]. Various activating and repressing histone modifications dictate gene transcription, and chromatin dysregulation can result in pathological cardiac gene expression.

Epigenetic mechanisms have been hypothesized to be a crucial interface between genetic and environmental factors to explain the theory of metabolic memory in diseases such as diabetes. Metabolic memory in diabetes focuses on the interrelationship between stressors (i.e., hyperglycemia, oxidative stress, chronic inflammation), which induce mechanisms that persist after the stressor is removed (i.e., controlled hyperglycemia) [8]. In a study by Brasacchio et al., even just transient exposure to hyperglycemia resulted in sustained activation of NF-κB (nuclear factor-kappa B), a key regulator of inflammatory gene expression, which may contribute to the later development of diabetic complications [9]. Similarly, it is well known that many cardiac injuries can predispose to future cardiac arrhythmias, and much work to date has focused on understanding this arrhythmogenic substrate at the tissue level, including tools to assess fibrosis, though the underlying cellular mechanisms are less well understood [10]. Clinically, it is well known that arrhythmias such as atrial fibrillation beget further atrial fibrillation, and perhaps a similar mechanism for electrical “remodeling” and arrhythmia predisposition may also be a crucial interface between genetic predisposition and acquired factors including age, hypertension, and diabetes. When these electrical changes are long-lasting after removal of the stressor, this “remodeling” may be best understood as cardiac “reprogramming.”

Our group has shown in a clinically relevant murine heart failure model, encompassed by progressive heart failure via transverse aortic constriction (TAC) plus small apical myocardial infarction, that Notch signaling is transiently reactivated in the adult heart, including reactivation in atrial and ventricular cardiomyocytes [11, 12]. Though activation of Notch within embryonic cardiomyocytes would lead to cardiomyocyte cell division, the effects in the adult heart are quite distinct. Instead of kick-starting a regenerative process, Notch activation in adult murine myocytes results in sustained alteration to ion channel gene expression and electrical currents which predisposes to arrhythmias [12]. It will be interesting to understand how this signaling process relates to ventricular arrhythmias which are frequently associated with heart failure. Indeed, Weinheimer et al. built on the clinically relevant heart failure mouse model described above and followed this with “debanding” to remove the stressor, thus restoring left-ventricular (LV) structure. However, LV function was only partially recovered and there was incomplete reversal of transcriptional changes associated with heart failure, which correlates with what is often seen clinically in heart failure patients [13]. Specifically, we see that after an initial heart failure diagnosis and following guideline directed medical therapy, some patients can partially recover cardiac function with resolution of heart failure symptoms. However, these patients often regress and are re-hospitalized, indicative of persistent mechanisms that trigger heart failure. Current work is focused on understanding the role of Notch signaling in this process, which we hope may ultimately result in better ways to predict which patients are most at-risk for life-threatening arrhythmias.

## Cardiac Chamber-Specific Histone Modifications and Electrophysiology

Embryologically, the left and right ventricles arise from the first and second heart fields, respectively, and are exposed to distinct signaling pathways during cardiac development [8]. This programming in the form of epigenetic “memory” may ultimately underlie some of the baseline electrical differences seen within various regions of the adult heart. A review by Molina et al. illustrates electrophysiological features and differences between the right ventricle (RV) and LV [14]. For example, the RV expresses lower levels of cardiac Na^+^ channels and higher levels of cardiac K^+^ channels when compared with the LV, which contributes to slower conduction in the RV [15]. Specifically, these differences can provide a critical substrate for developing life-threatening arrhythmias that originate in the right ventricle including the ventricular outflow tract in disorders such as Brugada syndrome, where inherited mutations of the gene that encodes Na^+^ channels (*SCN5A*) or the direct Notch signaling pathway target *HEY2* (Hairy/enhancer-of-split related with YRPW motif protein 2), among other mutations, can result in ventricular arrhythmias and sudden cardiac death [16, 17].

In addition to regulating baseline physiology, developmental programming may also dictate the differential response to stress in various regions of the heart, a response often referred to as reactivation of the “fetal gene program.” An early study to distinguish cardiac-specific epigenetic regulation of gene expression in normal murine hearts used chromatin immunoprecipitation (ChIP) analysis to compare LV versus RV expression levels of atrial natriuretic peptide (ANP) and brain natriuretic peptide (BNP). In normal wild-type mice, the group found increased levels of Histone 3 lysine 4 dimethylation (H3K4me2) and Histone 3 lysine 9 acetylation (H3K9ac) with concomitantly higher expression levels of ANP and BNP in the LV compared to RV [18]. In a separate study by Hohl et al., the group compared human non-failing and failing hearts where they showed upregulation of ANP and BNP in failing LV compared to non-failing LV without changes in H3K9ac or H3K4me2. However, there were reductions in the gene-silencing marks (H3K9me2 and H3K9me3), illustrating distinct mechanisms for gene regulation in the pathological state [19]. To this end, it would be interesting to study histone modifications in arrhythmogenic right ventricular cardiomyopathy where RV dysfunction predominates, given that there is greater upregulation of BNP in the RV compared with the LV in these patients [20]. These studies reveal maladaptive reactivation of fetal gene programs can occur in a chamber-specific manner and highlight that there may be distinct mechanisms for gene regulation via histone modification during homeostasis and after cardiac stressors [21].

**Fig. 1:**
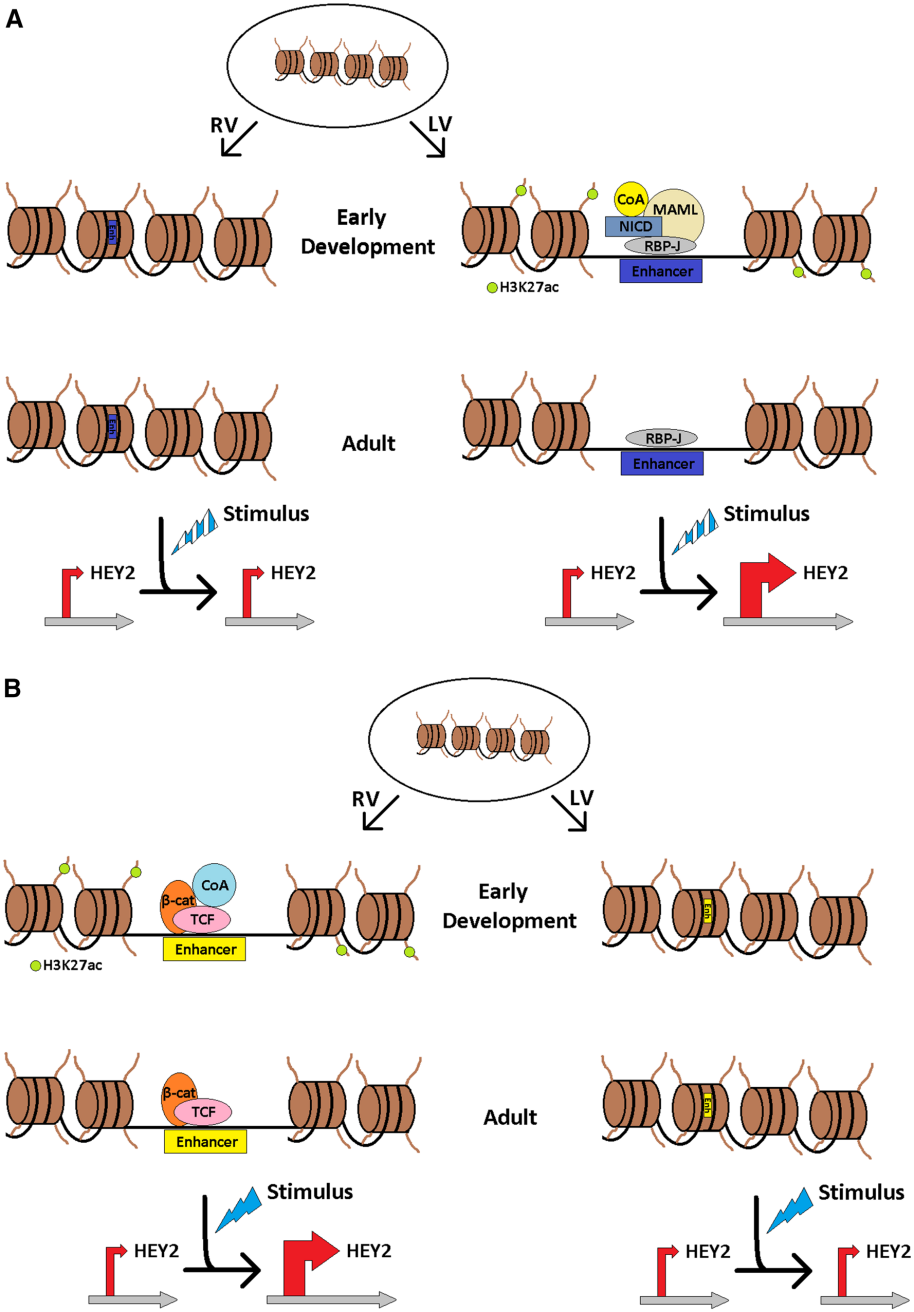
Schematic of chamber-specific “priming” and differential *HEY2* expression levels. **a** Canonical Notch signaling regulates early cardiac development mediated by nuclear translocation of the Notch intracellular domain (NICD), forming a complex with the DNA-binding transcription factor RBP-J (recombination signal binding protein for immunoglobulin kappa J region), MAML (mastermind-like protein), and other coactivators (CoA) to form the Mastermind-containing transcriptional complex at the enhancer region (blue). In the adult during homeostasis, RBP-J is bound to this blue enhancer element in the left ventricle (LV), but not the right ventricle (RV). Upon a pathologic stimulus that reactivates Notch signaling, expression of *HEY2* is upregulated only in the “primed” LV, whereas there is no change in *HEY2* expression levels in the RV. **b** Canonical Wnt signaling regulates early cardiac development that is mediated by nuclear translocation of β-catenin (β-cat) forming a complex with the DNA-binding T-cell factor (TCF) and other coactivators at a distinct *HEY2* enhancer region (yellow). In the adult during homeostasis, β-catenin is bound to the yellow enhancer in the RV, but not the LV. In reciprocal fashion to Notch signaling, a stimulus that reactivates Wnt signaling results in *HEY2* upregulation in the “primed” RV, but no change in *HEY2* expression in the LV

The Notch signaling pathway regulates multiple aspects of embryogenesis, from processes as diverse as cardiac structure to programming cardiac electrophysiologic properties [22]. We have previously shown that developmental Notch gain of function (GOF) mice have increased action potential duration (APD) in the LV [11]. Interestingly, in Notch-activated mice, *Hey2* expression levels are upregulated in the LV, whereas *Hey2* expression levels are downregulated in the RV. These gene expression changes are associated with increased Histone H3 lysine 4 trimethylation (H3K4me3) at the *Hey2* promoter in the LV, while there is loss of the H3K4me3 marks in the RV. Furthermore, the key transcriptional cofactor RBP-J (recombination signal binding protein for immunoglobulin kappa J region), is dynamically bound to the proximal *Hey2* promoter and also to an enhancer specifically within the LV but not in the RV [11]. Figure 1a shows a schematic of the “priming” of the LV that occurs during early cardiac development, which may later facilitate the expression of *Hey2* in adulthood following a specific trigger that induces Notch. Collectively, these results demonstrate that chamber-specific histone modifications can result in distinct electrical phenotypes and regulation of gene expression.

More recently, we demonstrate how embryonic programming via the Wnt signaling pathway also regulates electrophysiology in a chamber-specific manner [23, 24]. It has long been postulated that *Hey2* is regulated by mechanisms in addition to Notch signaling within the ventricles. We demonstrate for the first time that *Hey2* is a direct Wnt target only in the RV and not the LV, which may have relevance to our understanding of RV-specific arrhythmias [23]. Using both Wnt gain and loss-of-function approaches, additional genes regulating cardiac electrophysiology, *Gja1* (gap junction alpha-1, encodes connexin 43) and *Scn5a* (encodes Na_v_1.5 protein), were shown to be differentially regulated. Gene expression changes result in slower conduction velocity in both the Wnt gain and loss-of-function RV, but not LV, though the mechanism of slowed conduction is distinct in these models. Indeed, the chamber-specific gene expression changes predispose the mice to ventricular tachycardia with physiologic stimulation of the RV only. ChIP analysis of wild-type embryonic hearts demonstrates enrichment of the active H3K27ac mark in a *Hey2* enhancer region shown to be bound by the Wnt effector β-catenin in the RV only, while a distinct *Hey2* enhancer demonstrates H3K27ac enrichment in the embryonic LV. Since *Hey2* regulates important electrical properties within the RV, this example highlights how a chamber-specific understanding of embryonic transcriptional and epigenetic programming may help us to better understand arrhythmias which occur later in life. Figure 1b represents a model of the “priming” of the RV that occurs in early cardiac development, which may later facilitate the expression of *HEY2* in adulthood following a specific trigger where Wnt signaling is reactivated.

## Histone Modification in Cardiomyopathy

Two hallmark electrophysiological findings in failing hearts include conduction slowing and action potential prolongation. Electrophysiological heterogeneity within the heart presupposes the dysregulation of multiple key components of the action potential, including downregulation of repolarizing K^+^ currents (*I*_K1_, *I*_to_, *I*_Kr_, *I*_Ks_), alterations to depolarizing Ca^2+^ currents (*I*_Ca-L_) and Ca^2+^ transporters (NCX, SERCA2a), and downregulation of depolarizing Na^+^ currents (*I*_Na_). Connexin 43 is a key cardiac gap junction protein that can be downregulated or improperly distributed which also results in slowed conduction [25, 26].

Several studies have focused on the downregulation of repolarizing K^+^ currents, offering an illustrative example of the interplay between heart failure, electrophysiologic remodeling, and histone modifications. In the heart, *KCNIP2* (potassium channel interacting protein 2, which encodes the KChIP2 protein) interacts with the subfamily of voltage-gated potassium channel (Kv4) to increase current density, accelerate recovery from inactivation, and slow inactivation kinetics [27]. Collectively, changes in KChIP2 levels alters the fast component of the cardiac transient outward potassium current (*I*_to_) and contributes to APD prolongation and conduction slowing in both humans as well as canine models of heart failure [28, 29]. As discussed above, cardiac injuries and subsequent progression to heart failure reactivates Notch signaling in mouse models of heart failure, with concomitant downregulation of Kcnip2, and loss of H3K4me3 associated with dynamic RBP-J binding to the *Kcnip2* promoter [11]. Bolstering the significance of these findings, a study by Stein et al. demonstrated that loss of the same H3K4me3 methylation site through adult-inducible deletion of PAX-interacting protein 1 (PTIP), an essential cofactor for H3K4 methylation, resulted in reduced *Kcnip2* expression levels [30]. In addition, PTIP-deficient mice exhibited APD prolongation with reduced *I*_to_ and development of premature ventricular beats. Therefore, understanding how post-translational histone modifications are regulated is relevant to understanding cardiac electrophysiology, especially in the setting of heart failure.

Downregulation of *KCNIP2* has been shown to be a consistent finding across the spectrum of human cardiomyopathies. Exciting work recently demonstrates that KChIP2 protein not only contributes to *I*_to_, but it also regulates a transcriptional network in the heart. In a paper by Nassal et al., KChIP2 was shown to regulate microRNAs (miRNA) important for conduction and maintenance of electrical stability through KChIP2 binding to the miR-34b/c promoter region in the nucleus, supporting the notion that KChIP2 can behave as a transcriptional repressor [31]. In both neonatal rat cardiomyocytes exposed to phenylephrine as a mimic of neuro-hormonal overload and samples of human heart failure tissue, downregulation of KChIP2 and concomitant upregulation of miR-34b/c was observed. Restoration of KChIP2 expression levels not only reverted miR-34b/c levels back to baseline, but also restored channel function and impeded reentrant ventricular arrhythmias in a cell culture model. Given the importance of *KCNIP2* in regulating transcription and electrical remodeling in heart failure, understanding how it is regulated will be of clinical importance.

More broadly, several exciting studies offer a blueprint for understanding the role of epigenetics in heart failure. Studies by Rosa-Garrido et al. used genome-wide chromatin conformation capture (Hi–C) of isolated adult cardiomyocytes from a TAC heart failure mouse model to reveal alterations in chromatin compartmentalization and looping, demonstrating chromatin remodeling in conjunction with decreased enhancer interactions in heart failure [32]. To support these findings, the team generated a knock-out mouse of CTCF, a chromatin structural protein with key roles in regulating genome architecture and accessibility [33]. CTCF knock-out mice not only mirrored the chromatin remodeling findings of the TAC heart failure mice but also recapitulated the cardiac dysfunction seen in heart failure. As a clinical correlation, CTCF itself was found to be downregulated in end-stage heart failure patients requiring left-ventricular assist devices. Not surprisingly, CTCF knock-out mice did not reveal changes in H3K27me3 nor in H3K4me3, indicating the effects occurred via an alternative epigenetic mechanism to histone modifications and illustrating the critical role CTCF plays in chromatin regulation and heart failure.

## Conclusion

Signaling pathways active during cardiac development program cardiomyocyte identity. In the adult, these developmental pathways can be reactivated in disease states during pathologic remodeling where they can bind to chamber-specific enhancer and promoter regions and are associated with specific histone modifications. These mechanisms of gene regulation coordinate differential transcription of genes such as *HEY2* and *KCNIP2* in a chamber-specific manner, which may contribute to deadly ventricular arrhythmias.
